# Young people’s experiences of COVID-19 messaging at the start of the UK lockdown: lessons for positive engagement and information sharing

**DOI:** 10.1186/s12889-022-12755-3

**Published:** 2022-02-18

**Authors:** Sofia T. Strömmer, Divya Sivaramakrishnan, Sarah C. Shaw, Kathleen Morrison, Millie Barrett, Jillian Manner, Sarah Jenner, Tom Hughes, Polly Hardy-Johnson, Marike Andreas, Donna Lovelock, Sorna Paramananthan, Lisa Bagust, Audrey Buelo, Kathryn Woods-Townsend, Rochelle Ann Burgess, Nancy Kanu, Malik Gul, Tanya Matthews, Amina Smith-Gul, Mary Barker, Ruth Jepson

**Affiliations:** 1 MRC Lifecourse Epidemiology Centre, University of Southampton, Southampton General Hospital, Southampton, UK; 2 NIHR Southampton Biomedical Research Centre, University of Southampton and University Hospital Southampton NHS Foundation Trust, Southampton, UK; 3 Scottish Collaboration for Public Health Research and Policy, School of Health in Social Science, The University of Edinburgh, Edinburgh, UK; 4 Southampton Education School, Faculty of Social Sciences, University of Southampton, Southampton, UK; 5 Institute for Global Health, University College London, London, UK; 6 Wandsworth Community Empowerment Network, London, UK; 7 School of Health Sciences, Faculty of Environmental and Life Sciences, University of Southampton, Southampton, UK

**Keywords:** Young people, Government messaging, Pandemic, COVID-19, Qualitative, Adolescence

## Abstract

**Background:**

To reduce COVID-19 infection rates during the initial stages of the pandemic, the UK Government mandated a strict period of restriction on freedom of movement or ‘lockdown’. For young people, closure of schools and higher education institutions and social distancing rules may have been particularly challenging, coming at a critical time in their lives for social and emotional development. This study explored young people’s experiences of the UK Government’s initial response to the pandemic and related government messaging.

**Methods:**

This qualitative study combines data from research groups at the University of Southampton, University of Edinburgh and University College London. Thirty-six online focus group discussions (FGDs) were conducted with 150 young people (Southampton: *n* = 69; FGD = 7; Edinburgh: *n* = 41; FGD = 5; UCL: *n* = 40; FGD = 24). Thematic analysis was conducted to explore how young people viewed the government’s response and messaging and to develop recommendations for how to best involve young people in addressing similar crises in the future.

**Results:**

The abrupt onset of lockdown left young people shocked, confused and feeling ignored by government and media messaging. Despite this, they were motivated to adhere to government advice by the hope that life might soon return to normal. They felt a responsibility to help with the pandemic response, and wanted to be productive with their time, but saw few opportunities to volunteer.

**Conclusions:**

Young people want to be listened to and feel they have a part to play in responding to a national crisis such as the COVID-19 epidemic. To reduce the likelihood of disenfranchising the next generation, Government and the media should focus on developing messaging that reflects young people’s values and concerns and to provide opportunities for young people to become involved in responses to future crises.

## Background

In March 2020, the UK Government and devolved administrations introduced a first round of lockdown restrictions in response to the global COVID-19 pandemic. Public venues were closed. All unnecessary travel was prohibited. People could only leave their homes to shop for necessities, exercise once daily, to meet any medical need or to provide care or help to a vulnerable person. Initially, rules in Scotland and England were the same, co-ordinated under a UK four nations approach. The messaging was: “Stay at home, protect the NHS,^1^save lives”.

During this time, schools, colleges, and universities were closed and young people faced a protracted period of isolation, the impacts of which are not yet fully understood. For many, national exams were cancelled, causing anxiety about uncertain futures [1]. This disruption was experienced by a population who are in the midst of a critical period of development, involving major biological, psychological, social, and institutional transitions [2]. Increased need to be with peers, heightened sensitivity to social evaluation and influence and being inclined to take risks [3–5] together suggest that it might have been particularly difficult for young people to follow COVID-19 guidance that involved physical isolation and social distancing [6].

Being in a critical period for development means that experiencing trauma during adolescence may have specific and long-lasting consequences for young people [5]. The pandemic and the associated disruption to their normal lives may represent such trauma [7]. Disruption to education is likely to have consequences for young people’s economic opportunity in the medium term, and their employment, human rights, social capital and economic productivity in the longer term [8]. Further impacts of the pandemic response may well be seen on their health and potentially on the health of their future children, particularly through the changes it may have led to in their eating, sleeping and physical activity habits and the way those habits are carried into adult life [9, 10].

Some research suggests that young people may however demonstrate resilience in the face of the pandemic compared to other age groups. Recently published research shows that parents report smaller declines in the psychological wellbeing of adolescents, compared to children under 11 [11]. These differences may be elucidated by a recent study with younger children (7–11) which highlighted that these pre-adolescent children expressed sadness and fear of their family and friends being at risk of dying from COVID-19 [12]. In contrast, young people 12–17 years have reported that the greatest impacts of the pandemic have been disruption to their learning because of school closures and limited face-to-face interaction with their social networks [13]. Understanding young people’s psychological and behavioural responses to control measures and messaging is crucial to mitigating the effects of the pandemic [11].

The challenges imposed by the pandemic present an issue for all young people but will affect different groups of young people in different ways. The disparate impacts that the COVID-19 crisis has had on ethnic minority communities in the UK and across other high-income countries is already being seen [14–16]. The vulnerability of members of ethnic minority communities is not inherent; it is a product of structures and is reinforced through marginalisation from health, economic and additional social safety nets that should protect them [17]. Young people from ethnic minority communities are not just dealing with challenges that the pandemic represents for those at their stage of life, but are also managing the threat to their communities, given their increased vulnerability to infection and severe illness.

Researchers and public health authorities in the UK have not engaged directly with young people through the pandemic to understand their perspectives on the Government’s response or related messaging; a striking decision given debates that position young people as driving-up community infection rates [18]. This paper offers unique insights from young people on how they perceived government decisions and messaging at the start of the March 2020 lockdown, how they responded, and what they thought would make messaging more effective in reaching other young people.

## Methods

### Aim

This research aimed to answer the following questions:

- How did young people respond to UK government messaging early in the COVID-19 pandemic?
- How does this response inform the messaging aimed at young people during the rest of the pandemic and in future crises?

### Design

This exploratory qualitative study combined data from three larger studies, conducted independently but with similar methods and research aims, by the University of Southampton, and University College London (UCL), England and the University of Edinburgh, Scotland. Data collection was through online focus groups, transcripts of which were analysed using Thematic Analysis [19]. Ethical approval for these studies was received from the University of Southampton Faculty of Medicine Ethics Committee [Ethics Number: 56068], UCL Ethics Committee [Project ID 16,127/003] and the University of Edinburgh School of Health in Social Science Research Ethics Committee [Reference: STAFF182]. All aspects of all studies were performed in accordance with the relevant guidelines and regulations (e.g. Declaration of Helsinki). The reporting of these studies follows the consolidated criteria for reporting qualitative research (COREQ) [20]. Consultations were undertaken with 23 young people aged 12 to 16 in Southampton on March 30th 2020 to plan the design and conduct of this study. These consultations provided information about the key social media platforms that could be used to engage with young people in this study. As a result, groups of young people were communicated with using Snapchat and Discord to organise focus groups and for additional data collection later undertaken by Southampton researchers (see below). Focus groups were hosted on Zoom. These early consultations provided insights for the development of topic guides and clarified the acceptable length and frequency of focus group discussions with young people. Following these consultations, the Southampton team went on to undertake six additional waves of data collection using these platforms over the course of the first 12 months of the pandemic.

### Study participants and setting

In the Southampton and Edinburgh studies, a convenience sample of participants was recruited using a snowballing technique initiated through the research teams’ professional and personal connections to young people and young people’s groups. Project managers from the UCL study purposively sampled participants from racially minoritized groups from a youth engagement network within the study’s partner organisation, Wandsworth Community Empowerment Network. An online form was used to share information sheets and consent/assent forms with parents and adolescents who expressed interest in taking part. Informed consent was obtained from parents/guardians for participants under 16 years of age, as well as from the participants themselves. Consent was received directly from participants aged 16 years or older.

**Fig. 1:**
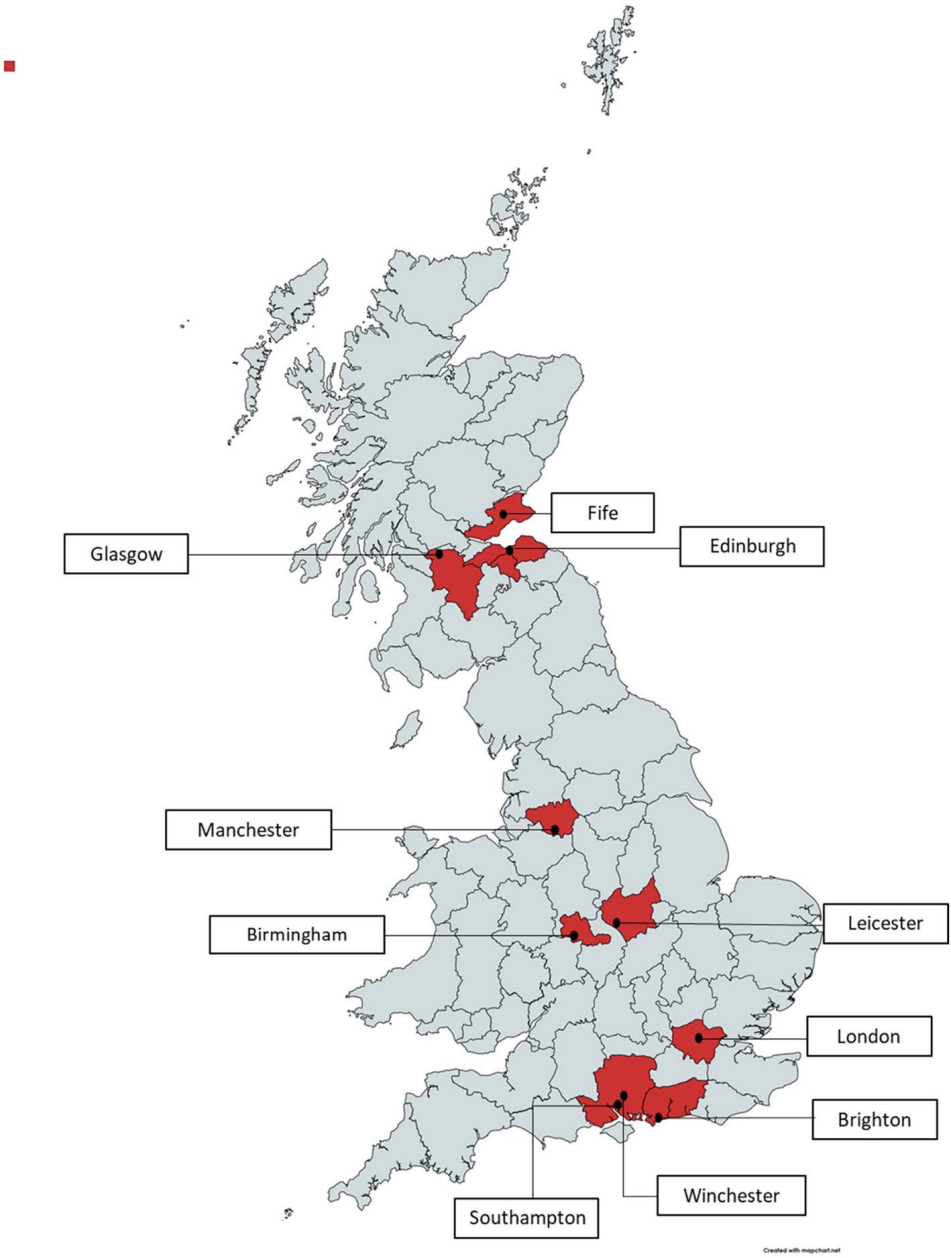
Geographical locations of participants

The Southampton sample included participants living in or near the English cities of Southampton, Winchester, Manchester, Brighton and Birmingham. The UCL sample included participants from areas in Greater London. The Edinburgh sample included participants from Edinburgh and the Lothians, Glasgow and Fife but included two participants from cities in England (c.f. map in Fig. 1).

### Procedure

The online focus group discussions (FGDs) reported in this paper were conducted between 6th April and 27th April 2020 by the Southampton and Edinburgh teams and between 7th July and 15th October 2020 by the UCL team. Additional focus groups have been conducted since by the Southampton team, and these findings will be reported elsewhere. FGDs were conducted using Zoom video conferencing software and were audio-recorded. Technical issues meant that one FGD was conducted using the chat function in Zoom. A copy of the chat transcript was saved and included in the analysis with transcripts from the audio-recorded focus groups. FGDs were led by a facilitator and an observer was present at each meeting. The Southampton FGDs were led by STS (post-doctoral research fellow), SCS (senior research assistant and PhD student), MB (senior research assistant), and PHJ (post-doctoral research fellow). The Edinburgh FGDs were facilitated by RJ (Professor), DS (post-doctoral research fellow), KM (research assistant and PhD student), AB (research assistant and PhD student), JM (research assistant), and TH (research assistant). All had previous experience of conducting qualitative FGDs. The UCL team conducted repeated FGDs with eight groups of 4–6 young people. Each group met three times resulting in 24 FGDs overall. FGDs were facilitated by RB (lecturer in Global Health), NK (research assistant), TM and ASG (partner organisation project managers), with the assistance of peer facilitators, who were members of the Wandsworth Community Empowerment Network.

FGDs lasted an hour and were facilitated using a semi-structured topic guide which asked about i) how the participants were spending their time, ii) changes to the participants’ lives since lockdown started, iii) participants’ views of the pandemic and the restrictions, including messaging about both and iv) ways in which young people might be involved in the response to COVID-19 (Table 1). The Southampton, Edinburgh, and UCL teams began their collaboration after the start of the projects; there are therefore some differences between the topic guides.

**Table 1:** Topic guides for focus group discussions held by Southampton, Edinburgh and UCL teams

Southampton topic guide	Edinburgh topic guide	UCL topic guide
**1. How are you spending your time?** - What has changed for you in the last week/2 or 3 weeks? - How have your eating habits changed? - How are you keeping active? - How have your feelings changed over the lockdown?	1. What is going on for you right now and how are you spending your time? 2. What has changed for you in the last few weeks or so? 3. How has it affected your studies or work? 4. How do you think people in your age group are dealing with this situation? 5. How important is community for you and what are your main sorts of communities? 6. What feelings of responsibility do you have if any in your community? 7. What have you been told about the coronavirus pandemic and who has told you this? Where did you hear about it? 8. What do you think about what you’ve heard and what you are being told to do? 9. How important do you think it is to follow the advice? 10. Which bit of advice do you find most difficult to do and why? 11. Which bits of advice do you find easiest to do and why? 12. What do you think we could do to help young people to stay safe and follow government advice? 13. What would the messages from the government have to contain to make young people follow the guidance? 14. Where should these messages be placed? 15. What could young people do that would help and how do we involve young people in keeping other young people safe?	**1. Introduction to project and general discussion of their experiences of lockdown period** - What is going on for you right now? How are you spending your time? What has changed for you in the last week/2 or 3 weeks? How are you coping? How have your feelings changed over the lockdown? -What are you doing to manage your time? What new things have you tried? **2. Focus on young people’s perceptions of policy responses and positive coping strategies** - How are you coping? How are you keeping active? - How do you think the government’s approach to lockdown impacted people? What do you think about the current messaging from the government and what you are being told to do? What would the messages from the government have to contain to make young people follow the guidance? Where should these messages be placed? - What could young people do that would help? How do we involve young people in helping their communities? **3. Development of bespoke programming messaging for young people from racialised groups to promote positive health behaviours and peer support during this global health crisis** - Was the lockdown well explained or justified by the government’s public health approach? Do you think that young people have received adequate or relevant information to justify the lockdown? - Do you know of any support resources that were available to young people during the lockdown? If so, what did you think of them? What would strong support services for young people look like? What support should be available to people as lockdown is eased? - What do you think of the current messaging for COVID-19? Is the messaging relevant? What is the messaging missing? What does the message include that has been useful or informative? What else would you want from the messaging & why? - Where do you think most people get their COVID-19 messaging or information from? Where do you get your COVID-19 messaging or information from? - What would an ideal public health message look like for young people like you?
**2. What are you doing to manage your time?** - What new things have you tried? **3. What do you think about the current messaging from the government and what you are being told to do?** - What would the messages from the government have to contain to make young people follow the guidance? - Where should these messages be placed? **4. What could young people do that would help? ** - How do we involve young people in helping their communities?		

### Analysis

Interview recordings were transcribed verbatim and thematic analysis was conducted following established methods [19]. Data was organised and managed for the Southampton team using NVivo software and Microsoft Word for the Edinburgh and UCL research teams. Coding of the Southampton and Edinburgh data was conducted by six researchers in each site (Southampton: STS, SCS, MB, SJ, DL and LB; Edinburgh: RJ, DS, KM, AB, JM and TH). The UCL data was coded by four researchers (RB, NK, MG and TM).

Data from the Southampton and Edinburgh research sites were coded deductively by the respective teams using an initial coding frame, developed by the Edinburgh team based on their topic guide. After the initial deductive coding process, the Southampton and Edinburgh teams used inductive coding to create new codes which described underlying meaning in the data. During the analysis process, these two research teams met online fortnightly to discuss similarities and differences between the two sets of data and respective coding, and the way in which the findings answered the research questions. The teams agreed on the key themes and sub-themes representing codes from each dataset and selected illustrative quotes to represent the meaning of each theme and sub-theme.

The UCL team coded their transcripts inductively and created codes that represented the underlying meaning in the data. The overlaps in and differences between the Southampton, Edinburgh and UCL data were then discussed by the research teams. The UCL findings were at this stage merged with the Southampton and Edinburgh findings to produce one collective set of insights.

#### Role of the funding sources

The funders were not involved in the study design, data collection, analysis or interpretation, writing of the report or the decision to submit the paper for publication.

## Results

### Participant characteristics

In total, 150 adolescents participated in 36 FGDs across the three studies (Southampton: *n* = 69 (FGD = 7), Edinburgh: *n* = 41 (FGD = 5), UCL: *n* = 40 (FGD = 24)). Table 2 outlines participant characteristics. Participants ranged from 11 to 25 years old (Southampton: 13–18 years, Edinburgh: 11–25 years, UCL: 16–25 years). Combining the studies, 61% of participants were young women (Southampton: 43.5% young women, Edinburgh: 73.2% young women, UCL: 80.0% young women). Ethnicity was missing for many of those in the Edinburgh sample, but based on observation of the focus groups was believed to be similar to the ethnicity profile of the Southampton sample with most participants being White British (61%), whilst the UCL sample was primarily Black African/Black Caribbean (87.5%).

**Table 2:** Participant characteristics

Characteristic	Southampton Focus Groups (*n* = 69 adolescents)	Edinburgh Focus Groups (*n* = 41 adolescents)	UCL Focus Groups (*n* = 40 adolescents)
Gender, *n* (%)			
Girl/Young woman	30 (43.5)	30 (73.2)	32 (80)
Boy/Young man	35 (50.7)	11 (26.8)	8 (20)
Missing	4 (5.8)	0 (0)	0 (0)
Age, *n* (%)			
11–14 years	11 (15.9)	11 (26.8)	0 (0)
15–18 years	51 (73.9)	14 (34.1)	19 (47.5)
19–25 years	0 (0)	16 (39.0)	21 (52.5)
Missing	7 (10.1)	0 (0)	0 (0)
Ethnicity, *n* (%)			
White British	42 (60.9)	7 (17.1)	0 (0)
Indian	5 (7.2)	-	3 (7.5)
Black African/Black Caribbean	3 (4.3)	-	35 (87.5)
Pakistani	2 (2.9)	-	0 (0)
Bangladeshi	1 (1.4)	-	0 (0)
Mixed	6 (8.7)	2 (4.9)	2 (5)
Other	6 (8.7)	3 (7.3)	0 (0)
Missing	4 (5.8)	29 (70.7)	0 (0)

### Findings

Seven themes were identified to describe the data. These are summarised below. Illustrative quotes are presented for each theme in Table 3. Data was analysed at FGD level, so no personal characteristics can be attributed to individual quotes. Pseudonyms are used throughout to protect the identity of the young people.

**Table 3:** Main themes, corresponding subthemes with illustrative quotes from each research team

Theme	Southampton subthemes	Southampton example quote	Edinburgh subthemes	Edinburgh example quote	UCL subthemes	UCL example quote
**1: Clearer and more consistent messaging was needed**	Agree that government messaging is working	“I’ve seen the message saying, ‘Stay at home’… they’ve been utilising hashtags like ‘protect the NHS’ and it’s on social media when we’re going through it…and we’re like… ‘Yeah, let’s do something about it’” *(Group 4, aged 17–18-years, Southampton)*	Short, simple messaging	“There’s not loads and loads of things you need to follow. They’re just sort of saying be careful and stay inside.” *(14–16 year old, Edinburgh)*	Aware of government messaging, but required research for understanding	“Um, I think when, like when Boris does his announcements, I think he should get someone else to go over what he's about to say. Like, because I feel like the team that he has around him, doesn't really know how to deliver the message properly. Because it takes a lot. When he first says something, I don't get it, like, I check online, and I see what other people like how they analyse what they *(the government)* said.” *(Group 6, 18–20-year-old, London)*
	Preference for clear, concise messaging	“I feel like in England… people are bending the rules and it’s not that clear, I’ve found, ‘cause I found at the start people like driving to exercise and like, and then people—people found out you got fines for it and I thought it was quite like mixed messages.” *(Group 5, 17–18-year-old, Brighton)*	Preference for short, simple messaging	“I think like putting it out in like short snappy messages rather than, you know, a big long spiel. Just having it real short, snappy slogans are easy to remember. Having it that way, you know, if you see it all the time you will end up just doing it automatically.” *(11–13 year old, Edinburgh)*	Preference for clear, concise messaging	“Everyone has no idea what's going on. People are scared. People are dying. So, the higher places like government, people aren’t sure as well. It makes it worse for everyone else. So, I understand where they’re coming from, but also they need to like set down rules, which will apply to you. Not just’Oh, you want to stay five feet apart? Or just stay six feet apart?’ It doesn't really make any sense.” *(Group 4, 16–17- year- old, London) *“I think the government just saying for us to just stay home. That's basically our role.” *(Group 3, 16–17-year-old, London)*
	Confusion around exam plans and poor communication	“The government weren’t really sure themselves what was gonna’ happen. And they were like, “Oh, yeah, in a week’s time you’ll find out.” …what grades… would be based on was quite a big question and to not to come out with that alongside the cancelling of the exams probably wasn’t the best idea, ‘cause it just left loads of people with unanswered questions.” *(Group 5, 17–18-year-old, Brighton)*	Ambiguous guidance Insufficient SQA communication At the beginning teacher’s advice differed from Gov advice Lack of clear information from universities Ambiguity around language: essential travel	“Since they announced that the exams were cancelled… you can tell the teachers have no idea what is going on. For some classes, the teacher is messaging the whole class every day with a different task. And some classes we have not heard anything since we were at school.” *(16–18-year-old, Edinburgh) *“They said there can only be essential travel. And then I wasn't sure… if I wanted to fly home… if that would be included….And then you got a letter from our university saying that going home was not essential travel. But then flights were still going” *(19–25-year-old, Edinburgh)*	Confusion around exam plans and poor communication	“I kind of want to know what they're thinking of, to help us with a future because of what has happened. Now a lot has changed, especially with the exams, and how we're going to be graded and everything.” (*Group 4, 16–17-year-old, London)*
**2: Positive messaging and messages with positive language were preferred**	Desire for positive messaging	“There has been so much focus on the bad things that are happening… but they haven’t really brought up what you could do with your time… it’s been a lot of ‘no, you can’t do this, no, don’t do that’ and maybe they need to look into what you can do instead.” *(Group 3, 15–16-year-old, Southampton)*	Preference for positive messaging Frustration at negative news Instagram used for positive stories	“Yeah, I feel like positive even draws you in more, because it reaches a point where you are like “no I don’t want to even look at that anymore” because you know that is going to be quite negative. I remember on the news the other day there was a really nice story reporting on people in a community doing something and we all wanted to watch it. The news had been on the whole evening and we hadn’t been kind of watching it, but when there is something nice it kind of attracts you more to the place it is coming from.” *(16–18-year-old, Edinburgh)*	Mitigated anxiety by avoiding negative messaging and news	“But when I got back to my own place, I think I think I had much more of a structured routine, and that really benefited me. And also, I don't have a TV. And I think that was such a benefit because I don't watch the news. So, I just felt less sort of anxious about what was going on. I wasn't seeing the death toll. So, it was sort of, not outside of mind completely, but it wasn't being constantly pushed in my face. Like, this is what's happening. And I think that helped a lot with like feeling anxious and stuff.” *(Group 1, 21–25-year-old, London)*
**3: Messaging should include young people and be on every available platform**	Messaging needs to be specific to young people Social media is the main source of information	“There’s actually been a lot on social media they are targeting quite a lot at our age on Twitter, on Instagram… on pretty much everything you see adverts for it all the time.” *(Group 4, 17–18-year-old, Southampton)* “Considering a lot of teenagers have become a lot more bound to technology, they could like spread the message [with] a viral video.” *(Group 2, 13–14-year-old, Southampton)*	Not relevant to 16 + age group Not acknowledging general struggles of young people	“I guess there is not that much information directed directly at us at the moment. So it would be good to have stuff that would be directly relating to us” (16–18-year-old, Edinburgh) “The sense of uncertainty also affects my motivation to try and figure out my future. I had intended to try and find a job for right after I handed in my dissertation, but I feel so unmotivated to do so and have convinced myself that finding a job will be impossible during this time” (19–25-year-old, Edinburgh)	Messaging needs to be targeted and accessible to young people Disconnection from traditional media outlets and seeking news from social media Inclusion is messaging is needed especially within racialised communities	“they (government officials) weren’t even referring to us when they were saying young people on the TV. They were referring to, like, people aged like 20 to 40ish. And they kind of excluded us in their, like plans and explanations, there hasn't really been much. I don't know, room to explain anything for us.” (Group 4, 16–17- year- old, London) “Every single news clipping I saw was screenshotted onto social media. Regardless, like young people don't read the news. It's the reason why a lot of people found out about social distancing, because it would be posted onto social media and suddenly it's like it's everywhere. It wasn't like all people have to watch news to know that we have the social distancing message measures or things like that. It was all over social media anyway.” (Group 7, 18–20-year-old, London)
**4: The government is the official source of information, but trust has been lost**	I don't fully trust the government to make the right decisions	‘I think they started it too late, because if they started in earlier, then we would have had less cases’ *(Group 7, 15–16-year-old, Birmingham)* ‘It’s kind of like an unnecessary loss of lives, like we shouldn’t have lost 20,000 people [….] if you act too late it’s going to have detrimental effects.’ *(Group 7, 15–16-year-old, Birmingham)* ‘I think they have done a really bad job, because they had their own idea of herd immunity [….] it turned out that idea was not a good one…’ *(Group 4, 17–18-year-old, Southampton)* ‘Testing is awful. The numbers are so low when we’re such a rich country, we can’t afford something like that, I think it’s a bad image really.’ *(Group 4, 17–18-year-old, Southampton)*	Trust in news depends on outlet Teachers as trusted authority but advice differed from gov advice at beginning Trust in the government Trust in social media: Twitter as a major/ preferred source	“They kind of all repeat each other. Like I’ve got BBC News and Sky News and Sun App on my phone. And so, in the morning I’ll get the notifications, and they’ve all said the same things, but the Sun’s added in some dramatic words.” *(14–16-year-old, Edinburgh)* “My teacher was saying we’re gonna not be off school… and then one day later… Boris Johnson and all the news and everything was saying all schools are closing on Friday and like that just made me trust the news more.” *(11–13-year-old, Edinburgh)* “I didn't really trust [the UK Government] from the beginning just because I felt like things should have been done and they weren't being done. So I was self-isolating even before the lockdown.” *(19–25-year-old, Edinburgh)* “Yeah I was trying to get…my information from official twitter accounts, you know like the First Minister twitter account and like following the sort of blue tick twitter accounts.” *(11–13-year-old, Edinburgh)*	Distrust and disagreement with the government’s handling	“Yeah. Exactly. There isn't as much transfer. And the government isn't, or hasn't been certain on how things are being transferred, or how to regulate it.” *(Focus Group 7, 18–20-year-old)*
**5: Non-compliance was viewed as being unfair and selfish**	I feel angry when I see others break the rules Influencers set a bad example Rebelling publicly Young vs old people adhere differently Adults and older people not following the advice Empathy and sympathy for others It would take something extreme to get people to listen to the advice	“Yeah, and it feels like when you see them going out, it’s a bit of a, like a bit of a kick in the face ‘cause you can’t go out and you’re following the guidelines and seeing like the massive numbers that come out on the radio of people that are dying every day and it’s like, I don’t know, I just find it really selfish that people are going out.” *(Group 6, 15–16-year-old, Manchester)* “People are doing it just to get a reaction, because me personally, I would never, ever go out and meet someone right now. I wouldn’t be allowed and I wouldn’t want to do it anyway. But if I did and if I put it on social media, I would expect people to give me a reaction because I know I’m not supposed to be doing it and so does everybody else…people do it, just so they can look to everybody else like, “Oh, they’re really cool.” ‘Cause they’re breaking all the rules and they’re going out to meet their friends when they know they shouldn’t be.” *(Group 6, 15–16-year-old, Manchester)* “I definitely think older people don’t listen as much because my grandparents, before they got ill were going out almost every day like on walks and stuff like. And then just socialising when they shouldn’t have. When I’ve gone round the shop to buy essential items, I do see a lot of middle-aged people out and about doing stuff and I just think like they should be staying at home, ‘cause the statistics do reflect that… but I definitely think that older people do go out more.” *(Group 4, 17–18-year-old, Southampton)* “Maybe they haven’t got the parental support and family support behind them. And they’ll just be saying “Whatever. I’m going out. I’m not staying home with my abusive dad.” I think it just depends where you’re from… I definitely think poorer communities probably will have it worse.” *(Group 4, 17–18-year-old, Southampton)* “I think the government should introduce like proper action against it to scare people from doing it in a way, like fines maybe ‘cause, yeah, it’s a bit annoying when people disobey the rules when everyone else is following it and it’s all in the best interest of the population to get rid of it.” *(Group 3, 15–16-year-old, Southampton)*	Perception of older age groups as non-compliant compared to peers Aware of the view that younger people did not take the restrictions seriously Perceptions of peers as “non-compliant” Perception of other people as “selfish” Perception that “others don't take measures seriously” ” Influencer behaviour Highlighting inequalities Empathy and sympathy for others	“And it’s just annoying, because if we’re young and we can do it, why can’t people that are older than us not follow the instructions.” *(Young person, aged 14–16, Edinburgh) *“I think that although lot of people are like the young people are not taking it serious enough and are still going out, I only know one person who is like considering going out. Everybody else has gone, no, we need to stay home, and I see more like going out to do things against the guidance in the old group.” *(14–16-year-old, Leicester) *“I've noticed though a lot of young people especially just don't care if they pass it on to someone who could die from it.” *(19–25-year-old, Manchester) *“It's kind of a shame seeing people not taking social distancing seriously because I see it as pretty selfish (maybe that's because I live at home with at-risk parents), but to not distance is not only putting yourself in danger but others close to you” *(19–25-year-old, Edinburgh) *“Where I live people are still like close and friends are chatting and stuff and I don’t think they are taking it too seriously.” *(11–13-year-old, Edinburgh) *“That you get the celebrities who do say it's so easy, like, why is no one doing it? Like come on, guys, just stay inside. And they've got their pool and their gym and, you know, like all their staff and just like these beautiful houses. And I think a lot of people's lives aren't take into consideration. Like people can be having really hard times at home and really struggle to be able to stay in the house even for a day.” *(19–25-year-old, Edinburgh) *“Staying inside isn’t easy for some but of course its a no brainer there’s nothing to really do in public spaces and I’m so aware of putting others at risk. I’m also lucky to have access to a garden” *(19–25-year-old, Edinburgh)*	Frustration towards those not staying home People meeting in large groups sets precedent for others to follow	“So, I just think that there's certain functions now where there's pubs open and people can turn to Brooks Park. I mean, I know Brooks Park do tickets, and you can be on regulation. I still think that people shouldn't really be having to go out in a large sum of people. You know, even I didn't want to get on the bus. Still. I still don’t want to get on the bus. I’d rather go to get the train. Yeah, but I don't think restaurants should be open right now.” *(Group 1, 21–25-year-old, London) *“So I will say that like, just from experiences from this entire lockdown period of me caring about people who have got sick, or people whose family have gotten sick and things like that, like, it's become a bit up and down for me because it's like you see large crowds gathering and then you don't hear about anything really happening.” *(Group 7, 18–20-year-old, London) “*It wasn't small. It was active. If you saw that beach, it was active. Like people were there, people were celebrating. This wasn't the first time this happened in Brighton when it was sunny. Everybody and their uncle was on that beach area. And it was disgusting. Nobody cared about COVID. And did we hear about any spikes of COVID? In Britain after any of these things happened? No. we didn’t hear nothing.” *(Group 7, 18–20-year-old, London) *“The park was pretty packed. You just see like the police just not even caring, like nonsense. Like no one's taking it seriously. It's like people not taking it seriously, influence other people not to take it seriously.” *(Group 3, 16–17- year- old, London)*
**6: A sense of responsibility to protect others drives compliance**	I'm happy to make sacrifices now if things can go back to normal quicker Behaviour of teenagers Having a positive attitude Bringing people together and a sense of camaraderie	“We all have an incentive because the more people stay at home…the sooner we can tackle this and the quicker we’ll be done with lockdown and we can get back to our own lives.” *(Group 4, 17–18-year-old, Southampton)* “I think it comes a lot from your parents because… if your parents are taking it more seriously, then it can encourage children to take it more seriously.” *(Group 6, 15–16-year-old, Manchester)* “I think the majority of people are listening to what they’re saying, especially in our age group and the five percent that aren’t… you see like people photographing sort of young people hanging out in a park …you can’t just assign a few small instances like that and [use it as] a ‘scape-goat’ for the government… “Oh, well, it’s not our fault. We did all we could. These people are hanging out in a park.” No, there are some things that you could’ve done better at the beginning.” *(Group 4, 17–18-year-old, Southampton)*	Things that make me want to comply Responsibility- prevent spread, protect NHS Fear for own personal safety and for friends and family To end lockdown/disruptions sooner Social pressure to follow or not follow advice Follow advice as they are told to do so by higher authorities	“There is a lot of responsibility, because obviously we are apparently the age group that if we carry it, it will be very mild and we are likely to pass it on without knowing.” *(16–18-year-old, Edinburgh)* “It’s not about us getting it, it’s about spreading it on to people who are more vulnerable and not crowding up the NHS.” *(11–13-year-old, Edinburgh)* “But I think if we all stay indoors or home, then it will be over quicker” *(16–18-year-old, Edinburgh)* “At the beginning of social distancing and gatherings I had to cancel plans and appeal to group chats not to have the meet-ups which I found challenging” *(19–25-year-old, Edinburgh)* “At the time I found it too much but after mentioning plans to parents they simply told me cancel it and spread the word so I did” *(19–25-year-old, Edinburgh)*	Concern for protecting others and responsibility to wider community Closely following social distancing guidelines Collective response by following guidelines Practical means to supporting community during this difficult time	“The new social norm has been putting your mask on and going outside and always wearing antibac and stuff like that. And before I didn't really care, but obviously, hanging around others, you have to be conscious and considerate. So now, of course, I do wear my mask. And I think about how it could affect others, as well as myself.” (*Group 7, 18–20-year-old, London) ***“**I haven't really been outside much. I haven’t been outside. I think, actually, I've only been outside I would say six times since before lockdown—Yeah, I hadn't really been outside.” *(Group 1, 21–25-year-old, London) *“Young people have done a lot, like they have helped. They have tried to do as much as possible with the limitations in place. It is like if we were to no go out, that would then be the issue. One thing that I want to point out is how young people responded to not having Carnival. Even though they were mad, this was the most mature I've actually seen young people in like in UK over something so big. Because Carnival has not been cancelled in ever since it's been going on.” *(Group 7, 18–20-year-old, London)*
**7: Young People want to do more to help others**	Helpful things you can do Feeling a sense of social responsibility to stay indoors Helping more vulnerable people Spreading the message about how to keep safe	“Yeah, I think most young people are doing the right thing. You know, staying at home, keeping a distance, sticking to your household. I think I’ve witnessed maybe one, maybe a few days ago where there was about fifteen boys, our age, playing football…and that was a bit alarming just to see, it was just like, you just think, ‘Why?’ But I haven’t seen many things to say that young people aren’t listening and I think young people could even be also like set—be set as like role models. Especially—my grandparents they just don’t seem to listen and we have to keep telling them, “Please, don’t go out unless you need to because you’re at risk.” And they just don’t seem to get it until you—we keep enforcing it and enforcing it, and it’s me and my brother who are the ones trying enforce it to them.” *(Group 4, 17–18-year-old, Southampton) *“I think just doing a bit of grocery shopping for some of my neighbours ‘cause they’re like elderly and in the vulnerable groups and stuff. Like that’s sort of helped me feel like I’m doing something at least ‘cause obviously we’re a bit young to do anything else I think.” *(Group 3, 15–16-year-old, Southampton) *“[Adolescents] could also spread awareness about social distancing. Because like everyone can get [COVID]. Considering a lot of teenagers have become a lot more bound to technology, they could like spread the message like make a viral video, so that old people will see.” *(Group 2, aged 12–13 year-old,, Southampton)*	Being a role model Educating others (friends, family & peers) More volunteer opportunities for under 18 YP More opportunities for university students YP organising themselves	“Well like if their friends say like, sometimes if they put it on their social media story ‘someone come out today’ they should say no don’t go out because you will pass it on.” *(11–13-year-old, Edinburgh)* “Yeah, my gran said she was fed up with it, and I was like ‘No, you need to stay home’ “*(14–16-year-old, Edinburgh)* “I think it would be good if there were more opportunities available for people under the age of 18 especially. Because I have looked into getting jobs, volunteering for the Red Cross, and other things but you have to be 18 to do all of them. So I feel like I am stuck in a stage where I can’t really do anything. But I am like young and fit, so I wish I was able to go out and do something.” *(16–18-year-old, Edinburgh)* “Totally, also uni students are a large part of the population the government could engage to help” *(19–25-year-old, Edinburgh)* “I can see quite a few young people organising themselves into volunteer groups to deliver groceries to vulnerable people, walk dogs etc.” *(19–25-year-old, Edinburgh)*	Call to highlight young people’s strength and contributions to their communities	“So if we kind of remove the idea that young people are useless, they don't do anything for our society, because a lot of the times older people just say that this generation is messed up, or this generation is like useless, or this generation, they just kind of put us down a lot. So we just end up seeing ourselves as just like, we're not helpful to society, there's no point in getting involved in anything. So, if we can just present ourselves and present young people as helpful that we need you like young people. ‘You're useful young people. We care about you, young people. You're special.’ Which is anything to be able to promote ourselves, and each other is just useful, we're not useless.” *(Group 3, 16–17- year- old, London)*

#### Theme 1: clearer and more consistent messaging was needed

Young people described doing their best to comply with government guidance on social distancing. During the early stages of the pandemic in April 2020, they felt that the overall messaging on social distancing was clear but that messaging about the indirect impacts of the virus on their lives, such as on their education and exams, was not clear. Young people wanted messaging that would make adhering to guidance actionable and straightforward.

*“Just having it real short. Snappy slogans are easy to remember. Having it that way, you know, if you see it all the time you will end up just doing it automatically.” (11–13-year-old, Edinburgh)*

Young people were not naïve and understood that there was uncertainty at every level of society, and that this made it difficult for the government to give clear answers and guidance on how people should respond to the situation. Young people from London, who were interviewed between July and October 2020, also felt, however, that the government's messaging lacked clarity and left young people feeling confused and fearful. They also felt that the language used for delivering messages was difficult to understand and could be improved.

*“Everyone has no idea what's going on. People are scared. People are dying. So the higher places like government, people aren’t sure as well. It makes it worse for everyone else.” (16–17-year-old, London)*

*“I think when Boris [referring to Boris Johnson, English Prime Minister during the pandemic] does his announcements…he should get someone else to go over what he's about to say… When he first says something, I don't get it… I check online, and I see how other people analyse what [the government] said.” (18–20-year-old, London)*

Participants felt that both government messaging to schools, and the communication from schools to students about issues such as exam plans was poorly managed. Many young people were left feeling anxious and stressed about their exams and the impact of decisions made on their grades and their futures.

*“The government weren’t really sure themselves what was gonna happen. What grades and stuff would be based on was quite a big question and to like not come out with that alongside the cancelling of the exams…just left loads of people with unanswered questions.” (17–18-year-old, Brighton)*

*“I kind of want to know what they're thinking of, to help us with a future because of what has happened. Now a lot has changed, especially with the exams, and how we're going to be graded and everything.” (16–17-year-old, London)*

University students also felt that there was a lack of clear guidance from universities in spring 2020 pertaining to assessments and felt that the language of travel-related government messaging was ambiguous.

*“They said there can only be essential travel. And then I wasn't sure… if I wanted to fly home… if that would be included…And then you got a letter from our university saying that going home was not essential travel. But then flights were still going” (19–25-year-old, Edinburgh)*

#### Theme 2: positive messaging and messages with positive language were preferred

Young people felt overwhelmed with constant streams of negative messaging and news focusing on daily death statistics and predictions of the terrible impact of the coronavirus on people, communities, and society. Young people wanted to see more positive messaging from government and other sources, including ideas for activities they could do during lockdown, rather than what often felt like an exhaustive list of things they could not do.

*“There has been so much focus on the bad things that are happening… but they haven’t really brought up what you could do with your time… it’s been a lot of ‘no, you can’t do this, no, don’t do that’ and maybe they need to look into what you can do instead.” (15–16-year-old, Southampton)*

Some young people responded to the distressing news coverage and media messaging by avoiding COVID-related media content altogether. This was a strategy adopted more commonly by young people from the London group who were interviewed later in the year, several months into the pandemic and prior to the second lock down in the UK.

*“I don't have a TV. And I think that was such a benefit because I don't watch the news. So, I just felt less sort of anxious about what was going on. I wasn't seeing the death toll… it wasn't being constantly pushed in my face.” (21–25-year-old, London)*

*“It reaches a point where you are like “no I don’t want to even look at that anymore” because you know that is going to be quite negative… on the news the other day there was a really nice story reporting on people in a community doing something and we all wanted to watch it.” (16–18-year-old, Edinburgh)*

Young people felt that the news and media coverage made them fearful, anxious and powerless in the face of the consequences of the pandemic and that life felt out of their control.

#### Theme 3: messaging should be aimed at young people and be visible on every available platform

Many young people felt they were not being prioritised in political decision making, and although they recognised that the COVID response was primarily designed to protect older and more vulnerable populations, they felt overlooked. In contrast to the younger groups for whom this was not really a point of discussion, the 16–18- and 19–25-year age groups felt particularly that their needs had not been addressed by Government information and that much of this information was not relevant to them. Despite the lack of adolescent-focused messaging, young people felt that they were reasonably well-informed about the situation, particularly in comparison with older people in the population, and disputed media claims that they were not engaging adequately or accessing information about COVID-19.

*“*[Government officials] *weren’t even referring to us when they were saying ‘young people’ on the TV. They were referring to, like, people aged like 20 to 40ish. And they kind of excluded us in their, like, plans and explanations. There hasn't really been much room to explain anything for us.” (16–17-year-old, London)*

Young people in the study suggested that the most effective way to communicate with them was through social media platforms such as Instagram, Twitter, TikTok or Snapchat. In contrast to those who felt that young people were being overlooked, some felt that these platforms had actually been used effectively for communication of Government messaging to their age group.

*“There’s actually been a lot on social media… they* [the UK government] *are targeting quite a lot at our age on Twitter, on Instagram… on pretty much everything. You see adverts for it all the time… spreading the message saying, “stay at home”. I mean they’ve been utilising hashtags as well, so like “protect the NHS”. (17–18-year-old, Southampton)*

Young people predominantly accessed news via their mobile phones rather than from television or radio, although some younger adolescents reported watching news with their parents for the first time during the pandemic. Young people also suggested communicating COVID related messages through physical on-street advertising, and local organisations such as schools, youth clubs, and sports clubs.

Young people from the UCL study, who were members of black and other minority ethnic (BAME) communities, expressed their concern about the quality of government messaging and how a lack of clarity could elevate the already heightened risk of the virus to members within their communities.

*“Just state what we need to do… They were talking about how BAME individuals are more susceptible to catching the virus or, like, there's a high death rate for BAME individuals… You know? Speak to BAME people! ‘Oh, whoa, this is a bit more dangerous for us’, which would, again, inspire us to obey the rules more often.” (16–17-year-old, London)*

Young people asked to be able to help shape the messaging for their age groups, but also for their cultural, ethnic and local communities. They also highlighted the need for tailored messaging, and for involvement of people who are being most impacted by the consequences of the pandemic.

#### Theme 4: the government is an official source of information, but trust has been lost

In the early days of lockdown, trust in information sources and decision-making by governments was a key factor in the way young people responded to the crisis. Mainstream news providers, such as the BBC (British Broadcasting Corporation) and Sky News, were trusted sources, while young people believed that tabloid newspapers were over-dramatising the crisis.

*“They kind of all repeat each other. Like I’ve got BBC News and Sky News and Sun App on my phone… and they’ve all said the same things, but the Sun’s added in some dramatic words.” (14–16-year-old, Edinburgh)*

*“And the government isn't, or hasn't been, certain on how things* [i.e. the COVID-19 virus] *are being transferred, or how to regulate it. And I just feel like things have been taken a bit out of proportion.” (18–20-year-old, London)*

Whilst younger adolescents were understandably less politically aware, older adolescents described a loss of trust in government decision-making through the early stages of the pandemic, primarily related to the belief that the March 2020 lockdown started too late. This led, in some cases, to some participants taking action independently of the Government advice.

*“I didn't really trust* [the UK Government] *from the beginning just because I felt like things should have been done and they weren't being done. So I was self-isolating even before the lockdown.” (19–25-year-old, Edinburgh)*

English participants also felt that death rates in England were unnecessarily high. They compared the situation in England to what they had seen and heard about other countries and felt that the UK government had fallen short. They felt that if the government had taken stronger action sooner by bringing in preventative measures and facilitated better capacity for testing, these losses could have been avoided.

*“It’s kind of, like, an unnecessary loss of lives. Like we shouldn’t have lost 20,000 people… Because we’re not, like, an unsanitary country. We have the facilities to sort of stop things like this happening, but if you* [i.e. the government] *act too late, it’s going to have detrimental effects.” (15–16-year-old, Birmingham)*

As described in Theme 3, a lack of inclusion of the views of young black people and their communities was blamed for the issuing of ‘blanket’ messaging, wherein non-specific sweeping statements about risks associated with COVID and the response that was required exacerbated a lack of trust already present amongst ethnic minority groups.

#### Theme 5: non-compliance was viewed as being unfair and selfish

There was a perception amongst young people at the start of the pandemic that it was actually older rather than younger people who were not following lockdown rules, despite the widely propagated view that it was the UK’s youth who were not complying. Some young people were concerned that their grandparents not taking the restrictions seriously. They also noted that some social media influencers set bad examples by violating rules and advertising their non-compliance.

*“And it’s just annoying, because if we’re young and we can do it, why can’t people that are older than us not follow the instructions.” (14–16-year-old, Edinburgh)*

*“The park was pretty packed. You just see like the police just not even caring, like nonsense. Like no one's taking it seriously. It's like people not taking it seriously influence other people not to take it seriously.” (16–17-year-old, London)*

Young people did also say that some of their peers were not complying with the rules and expressed anger and frustration about this. They felt it was unfair and selfish.

*“When you go out for walks you see people meeting friends, like it’s so obvious. Like our age, a bit younger, you see them and there’s people on Snapchat with friends and it’s just, it’s so bad.” (17–18-year-old, Southampton)*

On the other hand, young people also expressed compassion towards others who they thought might be in worse situations than themselves during the lockdown. They referred to their peers who might be living with abuse or in other challenging family situations and recognised that young people in those situations would be suffering disproportionately and would therefore be less able to comply with the restrictions on their movements.

*“Maybe they haven’t got the parental support and family support behind them. They’re the kids who are vulnerable to all these things. And they’ll just be saying “Whatever. I’m going out. I’m not staying home with my abusive dad.” I definitely think poorer communities probably will have it worse.” (17–18-year-old, Southampton)*

Young people felt that shock tactics and tailored messages highlighting the severity of the risk were needed to achieve behaviour change amongst those who were not complying. This view was at odds, however, with young people’s rejection of negative and overly ‘dramatic’ media coverage.

#### Theme 6: a sense of responsibility to protect others drives compliance

During these early stages of lockdown, young people showed an understanding of the importance of adhering to the government restrictions and many felt a sense of responsibility to protect others from the virus.

*“It’s not about us getting it, it’s about spreading it on to people who are more vulnerable and not crowding up the NHS.” (11–13-year-old, Edinburgh)*

Some were initially reluctant to cancel plans but did so after encouragement from parents and peers. Young people recognised that parental and peer attitudes towards the pandemic strongly influenced how likely they were to adhere to the restrictions.

*“I think that a lot of opinions that we have* [are] *actually not really the teen opinion. I think it comes a lot from your parents because your parents’ attitude… so if maybe your parents are taking it more seriously, then it can encourage your children to take it more seriously.” (15–16-year-old, Manchester)*

Many young people interviewed in early 2020 described the prospect of getting back to normal quickly as a key motivation for adhering to the government guidelines.

*“We all have an incentive because the more people stay at home…the sooner we can tackle this and the quicker we’ll be done with lockdown and we can get back to our own lives.” (17–18-year-old, Southampton)*

By autumn, mask wearing was the new social norm in the UK. Young people from London emphasised the need to be considerate and protect other people during this period.

*“The new social norm has been putting your mask on and going outside and always wearing anti-bac and stuff like that. And before I didn't really care, but obviously, hanging around others, you have to be conscious and considerate. So now, of course, I do wear my mask. And I think about how it could affect others, as well as myself.” (18–20-year-old, London)*

Young people emphasised that acting responsibly was not only about the big gestures, it was about doing the right thing when needed.

*“You don’t need to parade around doing up superman or whatever. You just need to pay attention and just open your eyes and think. If there’s a situation where you could help, are you going to go ‘Ah I’m too shy’ or ‘What if they tell me to get out of their face?’ or something. Just uh be a bit more aware and look to see if there’s any way you can help.” (18–20-year-old, London)*

For many young people, being responsible meant accepting that they would not be able to carry-out the plans they had to enjoy themselves over the summer; this included music festivals and particularly the Notting Hill Carnival for the young people in London. While they were sad and disappointed about this, they understood the rationale and accepted it.

#### Theme 7: young people want to do more to help others

In keeping with the sense of social responsibility described in Theme 5, young people believed they had a role to play in educating older people, such as parents and grandparents, about safety and the potential impact of COVID-19.

*“*[Young people] *could also spread awareness about social distancing. Because like everyone can get [COVID]. Considering a lot of teenagers have become a lot more bound to technology, they could spread the message like make a viral video, so that old people will see.” (12–13-year-old, Southampton)*

Young people were frustrated about being excluded from what they saw as a national effort and had suggestions about what they could do to help.

*“I think it would be good if there were more opportunities available for people under the age of 18 especially. Because I have looked into getting jobs, volunteering for the Red Cross, and other things but you have to be 18 to do all of them. So I feel like I am stuck in a stage where I can’t really do anything. But I am young and fit, so I wish I was able to go out and do something.” (16–18-year-old, Edinburgh)*

Adolescents in this study suggested that they could help vulnerable people by, for example, doing their grocery shopping, dog walking or volunteering in other capacities. Whilst some younger participants felt aggrieved that most volunteer roles were only for those aged 18 years and above, those aged over 18 years said that there were few opportunities for them as well. Some suggested that these were missed opportunities for voluntary organisations and for the Government to make use of young people’s energy and potential.

## Discussion

This qualitative study explored the impact on the lives of young people living in England and Scotland of messaging issued to support the UK Government’s initial response to COVID-19 and the restrictions on freedom of movement. Views from English and Scottish young people were similar. Unsurprisingly perhaps, younger adolescents were less politically aware than their older peers and were consequently less critical of the UK government response to the pandemic, including the COVID-related messaging. Over 18-year-olds from Edinburgh described experiences related to being at university and attempting to volunteer during the pandemic. Young people from London shared their experiences of being from ethnic minority communities who faced greater vulnerability to the impact of COVID-19. The findings from all the focus group discussions are presented below as they answer each research question.

### How did young people respond to UK government messaging early in the COVID-19 pandemic?

Young people felt strongly that important decisions about schools, exams and their social lives were made without any reference to them and their needs. They felt they had no control over decisions that were being made by government which fundamentally affected their lives whilst at the same time, being told that they were actually at lower risk of severe health consequences from the virus. For BAME communities the government messaging was even more confusing, in that whilst they were told their risk was higher, no guidance was given as to how members of these communities should respond to this information, how they could protect themselves and others in their communities, or what, if any, support was available to them. Young people suggested these factors led to many people feeling a sense of confusion or even helplessness which undermined their motivation to adhere to social distancing guidance.

Some previous research has suggested that adolescents are among the least adherent to public health guidelines [21]. This narrative was reflected in media coverage of the pandemic, which portrayed young people as rule breakers [18]. This is in contrast to the experience and opinions of adolescents who took part in focus groups in this study who reported complying with COVID-19 guidelines and expressed frustration about the behaviour of those who were not compliant. This was particularly directed at adults who they felt should have been setting a good example. Since these focus groups were conducted, a rapid qualitative interview study conducted with 21 UK young people early in the pandemic has been published. This group of young people also described working hard to comply with government public health guidelines and frustration with the public view that they were recklessly flouting the lockdown rules when the reality they experienced was that it was older generations who were doing this [18].

There was a disconnect between what messaging they thought would work for them or people like them (positive and encouraging), and what would work for “others” such as those openly flouting the rules (shock tactics). It is also possible that the young people who chose to participate in the focus groups were more likely to be compliant, or that participants may have overemphasised their compliance because that is what was socially desirable in the context of the focus group.

As a group also considered less at risk of being adversely affected by COVID-19, and with a lot of time and energy to expend, young people felt they had a lot to offer in terms of helping their communities. Young people were told that those aged under 18 years were unable to volunteer, but even those of age felt there were few opportunities to do this. In the months following the beginning of the first lockdown, volunteering rates among 16–24 year olds rose to 40% [22]. Even though formal opportunities seemed to be limited, young people expressed a strong sense of social responsibility and felt they could help in other small ways. Young people in this study suggested that older family members did not seem to recognise the severity of the situation and therefore they thought that young people could help by role-modelling safe behaviours and sharing what they knew about the purpose of social distancing. Previous research has shown that young people who are motivated to be healthy can positively influence the health behaviours of other family members through modelling and education [23]. It was evident from this study reported in this paper that young people wanted to feel confident that their efforts to comply with the guidelines were worthwhile and would help ensure a return to normal life. The ability to contribute and volunteer are indicators of young people's status as citizens and are known to affect how young people feel about themselves and their value in society [24]. Providing opportunities for young people to contribute in this way would have allowed young people to feel like valued members of society.

Some of the older participants described having low or diminished trust in government decision making. They did not believe that the social distancing measures set by the UK Government were sufficient nor introduced early enough. A recent Swiss study found that low trust in the government was a factor associated with non-compliance with COVID-19-related public health messages among young people [25]. The researchers argue that adopting behavioural changes may be perceived as burdensome if people do not trust in the authorities. Additionally, the pervasive nature of institutionalised racism and marginalisation undermines trust in government institutions and messaging for many ethnic minority communities [17, 26]. Building trust in the government may support young people’s compliance with prevention measures. The following sections outline potential ways in which young people could be meaningfully engaged with and their trust in government decision making and messaging enhanced.

### How does this response inform the messaging aimed at young people during the rest of the pandemic and in future crises?

Young people in this study called for the use of tailored, straightforward and simple messaging in public health campaigns. This is in line with previous research highlighting the appeal of such strategies [27]. Recommendations specify that, to be effective, messaging strategies should be positively framed and should emphasise the importance of adhering to guidelines in order to protect others [28]. Other research suggests, however, that positively framed health messages may be no more persuasive than the negative ones that young people rejected [29]. A shift of focus away from what they should not do, towards productive activities that they could do was suggested by young people as a strategy for improving their wellbeing, sense of control over their lives and morale. Young people suggested a dual strategy recognising that not all young people are the same nor equally compliant; they recommended highlighting of risk and the severity of consequences for those who were non-compliant and providing encouraging messages for those who were. The effectiveness of this dual strategy remains to be established.

There was significant negative media attention focused on young people and their compliance with lockdown rules during the early part of the pandemic. This may have been counter-productive for young people since evidence suggests that people are more likely to cooperate when they believe others are also cooperating [30]. Government leaders and the media should focus on reinforcing the message that adhering to guidance and restrictions is the right thing to do and that most people are trying to do so [28]. Rather than presenting a negative portrayal of young people and chastising them, it may be more constructive to instead include them in generating the solution, including publicising examples of the many contributions they are making to the crisis. There have been multiple calls for the engagement of strategic community actors in planning public health responses during COVID-19 drawing on lessons from previous pandemics [25, 31, 32]. Such strategies should also seek to engage young people as experts in their own right.

Recommendations for meaningful engagement with and effective messaging to young people in future emergencies based on the findings of this study are offered in Table 4.

**Table 4:** Recommendations for effective and inclusive messaging for young people

Recommendation	Strategies
**i) Involve and consult young people at all stages of planning and decision making about communication and messaging for their age groups**	• Set up a Youth Advisory Group as a key stakeholder in any national pandemic planning unit so that young people are represented at all stages of the process
	• Engage with Youth Parliaments and other national networks representing young people
	• Ensure engagement with a broad and diverse range of young people including marginalised or disenfranchised groups
	• Ensure appropriate channels of communication are used, including social media, and consider how to reach marginalized or disenfranchised groups (e.g. reaching out via community groups, faith leaders, youth workers)
**ii) Recognise how young people are influenced and how they influence others**	• Understand the role of influencers and people in positions of authority whose behaviour can influence and guide young people’s decisions. Identify who these people are early on and involve them in the communication with young people • Understand the importance of peer group behaviour in adolescence, and consider ways to use this positively and creatively
**iii) Include young people in constructive ways to help their communities**	• Set up systems whereby young people can volunteer locally to deliver food or other essential items to more vulnerable members of their community
	• Facilitate young people to connect with older people who may be more likely to suffer loneliness related to isolation, exacerbating the long-term consequences of the pandemic
**iv) Consider the diversity of young people and do not expect them all to react in the same way to messages or actions**	• Take context into account when shaping public health messages by adopting community focused practices, for example by partnering with community organisation
	• Create an inclusive and comfortable environment for young people to feel able to share their views honestly, for example by using peer-facilitators
	• Ensure that people from diverse social and cultural communities are represented in consultations and development of messaging and support services
	• Identify who trusted sources of authority are for different groups of young people, by asking the young people
	• Work with these trusted sources to develop communication strategies that are more likely to be effective
**v) Recognise the importance of positive messaging towards young people and their behaviour**	• Emphasise what young people can actively do to reduce the spread of the virus and harness these groups as agents of change
	• Recognise the role of mainstream media and it’s portrayal of young people. Seek to counteract negative media that unfairly portrays young people as ‘rule breakers’
	• Leaders should acknowledge the disproportionate impact of restrictions on young people and address groups within this population (e.g. school pupils, university and college students) directly and frequently. Leaders should also motivate young people’s sense of social responsibility and encourage them to act accordingly to collectively help prevent the spread of the virus

### Strengths and limitations

This study reports findings from an analysis of a unique, large combined qualitative dataset generated through conversations with diverse young people across the UK held at the beginning of the COVID-19 pandemic. Though the group represented diverse ages and ethnicities, and despite focused efforts, there were few young people from low socioeconomic status backgrounds in this study. This may be partly due to the snowballing recruitment strategy used, although the direct benefit of this recruitment strategy is that there was pre-existing good rapport within groups of participants which created a relaxed environment for honest and open discussion, and which facilitated the generation of rich and nuanced data. As the three research teams established their collaboration once data had already been collected, the Southampton, Edinburgh and UCL research aims and topic guides differed from one another in some ways. Additionally, based on Southampton and Edinburgh and the subsequent UCL data collection timeline, the salience of discussion topics changed between data collection periods. Any impact of this on the analysis was mitigated by the development of a co-created coding framework between Southampton and Edinburgh, and regular meetings between the three research teams to develop a unified approach to the reporting of findings.

## Conclusions

In the early phase of the pandemic, young people felt largely ignored by government messaging about issues that they felt directly affected them and their communities. Negative media portrayals of young people seemed at odds with the experiences that young people themselves were reporting. Young people caught up in the pandemic face a unique set of issues, the consequences of which are likely to affect their future lives and careers. Young people participating in this study felt none of these issues were being acknowledged or addressed. Despite being cut off from their social networks and mostly confined to their homes, young people still wanted to be able to help others and contribute to a societal effort. Young people are our future. If we want compliance and responsible behaviour from them, it behoves those in power to recognise, respect and enable young people to contribute to combating the crisis. Better engagement of young people from the outset may improve outcomes for the whole population whilst also limiting long-term negative effects on young people themselves. Governments would benefit from valuing the input of young people and harness the energy, dynamism, creativity, and commitment they bring.

## Data Availability

Anonymised participant data in the form of transcript text coded under each main theme and subthemes are shared after de-identification with researchers who provide a methodologically sound proposal. Proposals should be directed to ss3@mrc.soton.ac.uk; to gain access, data requestors will need to sign a data access agreement.

## Abbreviations

NHS: National Health Service (UK)
UCL: University College London
FGD: Focus group discussion

## Footnotes

1 National Health Service (UK).

## References

[1] Lee J (2020). Mental health effects of school closures during COVID-19. The Lancet Child & Adolescent Health.

[2] Blakemore S-J, Mills KL (2014). Is adolescence a sensitive period for sociocultural processing?. Annu Rev Psychol.

[3] Cardoos SL, Suleiman AB, Johnson M (2017). Social status strategy in early adolescent girls: Testosterone and value-based decision making. Psychoneuroendocrinology.

[4] Kilford EJ, Garrett E, Blakemore S-J (2016). The development of social cognition in adolescence: An integrated perspective. Neurosci Biobehav Rev.

[5] Fuhrmann D, Knoll LJ, Blakemore S-J (2015). Adolescence as a Sensitive Period of Brain Development. Trends Cogn Sci.

[6] Andrews JL, Foulkes L, Blakemore S-J (2020). Peer influence in adolescence: Public-health implications for COVID-19. Trends Cogn Sci.

[7] Dalton L, Rapa E, Stein A (2020). Protecting the psychological health of children through effective communication about COVID-19. The Lancet Child & Adolescent Health.

[8] Jimenez E. World Development Report 2007: Development and the next generation. Washington: World Bank Publications; 2006.

[9] Viner RM, Ross D, Hardy R, Kuh D, Power C, Johnson A, Wellings K, McCambridge J, Cole TJ, Kelly Y, Batty GD. Life course epidemiology: recognising the importance of adolescence. J Epidemiol Community Health. 2015;69(8):719-20. 10.1136/jech-2014-205300 PMC4515995 25646208

[10] Patton GC, Olsson CA, Skirbekk V (2018). Adolescence and the next generation. Nature.

[11] Waite P, Pearcey S, Shum A (2021). How did the mental health symptoms of children and adolescents change over early lockdown during the COVID-19 pandemic in the UK?. JCPP Advances.

[12] Thompson J, Spencer G, Curtis P (2021). Children's perspectives and experiences of the COVID-19 pandemic and UK public health measures. Health Expect.

[13] Fisher H, Lambert H, Hickman M (2021). Experiences of the coronavirus disease-19 (COVID-19) pandemic from the perspectives of young people: Rapid qualitative study. Public Health in Practice.

[14] Aldridge RW, Lewer D, Katikireddi SV, Mathur R, Pathak N, Burns R, Fragaszy EB, Johnson AM, Devakumar D, Abubakar I, Hayward A. Black, Asian and Minority Ethnic groups in England are at increased risk of death from COVID-19: indirect standardisation of NHS mortality data. Wellcome open research. 2020;5. 10.12688/wellcomeopenres.15922.1 PMC7317462 32613083

[15] Razai MS, Kankam HK, Majeed A, Esmail A, Williams DR. Mitigating ethnic disparities in covid-19 and beyond. bmj. 2021;372. 10.1136/bmj.m4921 33446485

[16] El-Mohandes A, Ratzan SC, Rauh L, Ngo V, Rabin K, Kimball S, Aaron B, Freudenberg N. COVID-19: a barometer for social justice in New York City. American Journal of Public Health. 2020;110(11):1656-8. 10.2105/AJPH.2020.305939 PMC7542288 32910679

[17] Burgess RA, Osborne RH, Yongabi KA (2021). The COVID-19 vaccines rush: participatory community engagement matters more than ever. The Lancet.

[18] ITV. ‘Lower compliance’ to lockdown among young, warns Michael Gove [Internet]. London: ITV; 2020 Apr 04 [cited 2022 Feb 16]. Available from: https://www.itv.com/news/2020-04-04/lower-compliance-to-lockdown-among-young-warns-michael-gove.

[19] Braun V, Clarke V (2006). Using thematic analysis in psychology. Qual Res Psychol.

[20] Tong A, Sainsbury P, Craig J (2007). Consolidated criteria for reporting qualitative research (COREQ): a 32-item checklist for interviews and focus groups. Int J Qual Health Care.

[21] Dixon H, Rayner, G. Parents of teenagers who flout coronavirus lockdown rules should be fined, police told. The Telegraph [Internet]. 2020 Apr 02 [cited 2022 Feb 16]. Available from: https://www.telegraph.co.uk/news/2020/04/02/parents-teenagers-flout-coronavirus-lockdown-rules-should-fined/.

[22] Roche M. Are young people replacing older people as the key volunteering group? [Internet]. London: nfpSynergy, 2020 - [cited 2022 Feb 16]. Available from: https://nfpsynergy.net/blog/are-young-people-replacing-older-peoplekey-volunteering-group.

[23] Lenne RL, Joyal-Desmarais K, Jones RE (2019). Parenting styles moderate how parent and adolescent beliefs shape each other's eating and physical activity: Dyadic evidence from a cross-sectional, US National Survey. J Exp Soc Psychol.

[24] Smith N, Lister R, Middleton S (2005). Young people as real citizens: Towards an inclusionary understanding of citizenship. J Youth Stud.

[25] Nivette A, Ribeaud D, Murray A (2021). Non-compliance with COVID-19-related public health measures among young adults in Switzerland: Insights from a longitudinal cohort study. Social Science & Medicine.

[26] Fekete L (2018). Lammy Review: without racial justice, can there be trust?. Race & Class.

[27] Randolph W, Viswanath K (2004). Lessons learned from public health mass media campaigns: marketing health in a crowded media world. Annu Rev Public Health.

[28] Van Bavel JJ, Baicker K, Boggio PS (2020). Using social and behavioural science to support COVID-19 pandemic response. Nat Hum Behav.

[29] Akl EA, Oxman AD, Herrin J, Vist GE, Terrenato I, Sperati F, Costiniuk C, Blank D, Schünemann H. Framing of health information messages. Cochrane Database of Systematic Reviews. 2011(12). 10.1002/14651858.CD006777.pub2 PMC12926860 22161408

[30] Ellemers N, van den Bos K (2012). Morality in groups: On the social-regulatory functions of right and wrong. Soc Pers Psychol Compass.

[31] Hargreaves J, Davey C, Auerbach J (2020). Three lessons for the COVID-19 response from pandemic HIV. The Lancet HIV.

[32] Richards P. What Might Africa Teach the World? Covid-19 and Ebola Virus Disease Compared. African Arguments [Internet]. 2020 Mar 17 - [cited 2022 Feb 16]. Available from: https://africanarguments.org/2020/03/what-mightafrica-teach-the-world-covid-19-and-ebola-virus-disease-compared/.

